# Sex Differences in Early Stages of Cardiorenometabolic Syndrome in Young Adults

**DOI:** 10.7759/cureus.93900

**Published:** 2025-10-05

**Authors:** Albina V Sineglazova, Guzel R Sadykova, Swapnil D Parve

**Affiliations:** 1 Department of Primary Care & General Practice, Kazan State Medical University, Kazan, RUS; 2 Internal Medicine, Parve Nursing Home, Buldhana, IND

**Keywords:** adult echocardiography, cardiorenometabolic syndrome, cystatin c, kidney dysfunction, metabolic risk factors, sex differences

## Abstract

Introduction: Cardiorenometabolic diseases represent a significant global health burden. Data highlight an alarming rise in cardiorenometabolic risk factors, such as adiposity, arterial hypertension, insulin resistance, elevated body mass index (BMI), dyslipidemia, and early renal dysfunction among adults. However, studies evaluating the sex-stratified burden, especially in young adults, are limited. The aim of this study was to investigate the differences between sexes in the early stages of cardiorenometabolic syndrome in young adults.

Methods: This cross-sectional study included 169 patients (89 female and 80 male participants). We conducted patient interviews along with comprehensive laboratory and instrumental analyses. Studied cardiorenometabolic parameters included anthropometry, body composition analysis, blood pressure measurements, lipid, glycemic and renal profiles, and factors constituting residual risk and echocardiographic parameters. Non-parametric statistical tests were performed.

Results: The median age of participants was 34 (30-39) years. Abdominal obesity, hyperleptinemia, elevated high-sensitivity C-reactive protein (hsCRP) and N-terminal pro-brain natriuretic peptide (NT-proBNP) levels, and elevated heart rate were more common in female participants. Male participants exhibited a higher frequency of elevated systolic and diastolic blood pressure (BP). Furthermore, hypertriglyceridemia, hyperuricemia, elevated cystatin C levels, and urine albumin to creatinine ratio were significantly more common in male participants. A similar trend was observed while evaluating the median values. Stratification by sex and cystatin C tertiles revealed that in women, higher cystatin C tertiles were associated with increasing BMI, waist circumference (WC), visceral fat, and glucose, hsCRP, and leptin levels. Notably, a statistically significant decline in estimated glomerular filtration rate (eGFR), based on the Chronic Kidney Disease Epidemiology Collaboration (CKD-EPI) cystatin C formula (eGFR_cysC_), was observed in both female and male participants. However, apart from this finding, no significant trends were observed among male participants. Key sex-specific significant interactions were identified for BMI, WC, waist-hip ratio, body fat percentage, visceral fat level, BP, high-density lipoprotein cholesterol, uric acid, eGFR_cysC_, hsCRP, leptin, NT-proBNP, and heart rate. Correlation analysis revealed that eGFR derived from cystatin C in female participants and creatinine in male participants demonstrated stronger and more extensive correlations with cardiometabolic and echocardiographic parameters than their respective individual biomarkers. The regression models further clarified these relationships. In female participants, eGFR based on cystatin C was independently predicted by hsCRP, visceral fat, systolic BP, and insulin. In male participants, eGFR based on creatinine was inversely associated with visceral fat level and positively associated with leptin.

Conclusion: This study underscores profound sex differences in cardiorenometabolic risk profiles among young adults, with female participants exhibiting adiposity-driven inflammation and male participants displaying early renal and vascular perturbations. While cystatin C and creatinine emerged as sensitive markers of cardiometabolic-renal crosstalk, cystatin C-based eGFR and creatinine-based eGFR better captured risks in females and males, respectively.

## Introduction

Cardiorenometabolic diseases such as coronary artery disease, congestive heart failure, atrial fibrillation, type 2 diabetes mellitus, chronic kidney disease, and others represent a significant global health burden and contribute to morbidity and mortality across all age groups [1,2]. The early stages of cardiorenometabolic syndrome are characterized by adiposity, and the presence of metabolic risk factors [2]. Emerging evidence highlights an alarming rise in cardiorenometabolic risk factors, such as adiposity, arterial hypertension, insulin resistance, elevated body mass index (BMI), and dyslipidemia, among young adults [3,4]. The literature suggests that these conditions are interconnected through shared pathways, including chronic inflammation, oxidative stress, and endothelial dysfunction, which collectively result in the cardiovascular-kidney-metabolic syndrome and contribute to end-organ damage [2]. However, the mechanisms driving these associations remain poorly understood, especially in younger individuals without overt disease.

A critical gap in the current research lies in the exploration of sex-specific differences in cardiorenometabolic risk profiles. Biological and hormonal variations between the sexes influence adiposity distribution, lipid metabolism, and vascular remodeling, yet studies often aggregate data or focus on older cohorts with established comorbidities. For instance, men typically exhibit higher rates of visceral adiposity and hypertension, whereas women are predisposed to subcutaneous fat accumulation and leptin dysregulation [5-7]. Such disparities underscore the necessity of sex-stratified analyses to unravel the distinct pathophysiological pathways.

Researchers are increasingly focusing on early-stage renal dysfunction within the context of cardiorenometabolic syndrome. Cystatin C serves as a sensitive renal function indicator [8], yet its role in cardiorenometabolic risk stratification, particularly among young adults, remains underexplored. Despite evidence suggesting hormonal influence on cystatin C production and clearance, sex-specific interactions between cystatin C and metabolic parameters are insufficiently studied [8]. Based on these observations, we designed our analysis to evaluate whether cystatin C and creatinine provide differential insights into cardiorenometabolic crosstalk in young women and men. To address the gap in sex-stratified data among young adults, this study aimed to investigate the differences between sexes in the early stages of cardiorenometabolic syndrome, and to evaluate whether cystatin C and creatinine provide distinct insights into cardiorenometabolic risk profiles in females and males, respectively.

## Materials and methods

Study setting, design, and participants

This cross-sectional study was conducted at an outpatient clinic affiliated with the Department of Primary Care and General Practice of Kazan State Medical University. We recruited 169 young adults (89 female and 80 male participants) aged 25-44 years. This age group is epidemiologically significant as it represents a critical window for the initial emergence and subclinical interaction of cardiorenometabolic risk factors. Investigating this cohort allows for the identification of early pathophysiological pathways prior to the confounding influence of established disease, offering valuable insights for primary prevention. The study was conducted between 2021 and 2022. A two-stage random sampling method was utilized to ensure representativeness and reduce selection bias. Initially, an outpatient clinic was randomly chosen by entering the names of all primary care centers, in Kazan, into a randomization tool. Following the selection of the primary care center, study participants were randomly selected using their medical record numbers. This strategy aimed to obtain a representative sample and minimize potential biases in the selection process.

Inclusion and exclusion criteria

The patient recruitment was done considering the criteria outlined in Table 1.

**Table 1:** Inclusion and exclusion criteria for participant selection

Inclusion criteria	Exclusion criteria
Participants aged 25-44 years	Patients with mental health conditions that interfered with the interview process
Participants who provided voluntary informed consent to participate	Patients with confirmed cardiometabolic disorders (including type 2 diabetes mellitus, coronary artery disease, congestive heart failure, atrial fibrillation, and chronic kidney disease)
	Patients with antiphospholipid syndrome and autoimmune inflammatory conditions
	Patients with confirmed cancer diagnoses
	Patients with decompensatory states of coexisting illnesses or conditions (such as liver or kidney disease)
	Patients with acute infections, endocrine disorders
	Patients with endocrine disorders
	Patients with other diseases or conditions that secondarily cause obesity
	Patients with presence of medical implants (e.g., pacemakers, silicone implants, or metal prostheses)
	Women who were pregnant or breastfeeding

Data collection

During the patient interviews, details such as demographics, anthropometry (height, weight, BMI, waist circumference, or WC, and waist-to-hip ratio, or WHR), and clinical data (blood pressure, or BP, oxygen saturation, and medical history) were recorded. Physical examination was performed according to guidelines. Weight and body composition were evaluated using bioelectrical impedance with a Tanita BC-601 body composition monitor (Tanita Corporation, Japan). The levels of body fat percentage and muscle mass of each segment were recorded as low, standard, and high. A visceral fat level rating in the range of 1-12 was considered normal, whereas 13-59 was recorded as excess visceral fat [9]. BMI was calculated and categorized according to the World Health Organization recommendations [10].

The workup was performed using fasting venous blood samples. The samples were collected using standard venipuncture techniques, and blood was drawn into appropriate collection tubes. Lipid profile analysis was performed using a Beckman Coulter AU480 automated chemistry analyzer (Beckman Coulter Inc., Brea, USA). Total cholesterol, triglyceride, high-density lipoprotein cholesterol (HDL-c), and low-density lipoprotein cholesterol levels were measured using an enzymatic colorimetric method. Non-high-density lipoprotein cholesterol (non-HDL-c) levels were calculated by subtracting HDL-c from total cholesterol. Fasting plasma glucose levels were measured using the hexokinase assay. An oral glucose tolerance test was performed by giving a 75 g glucose load and measuring two-hour post-load glucose. Glycated hemoglobin (HbA1c) was measured by an automated immunoturbidimetric test using Randox RX series kits (Randox Laboratories Ltd., United Kingdom). Insulin levels were measured by a chemiluminescent immunoassay using a Siemens Immulite 1000 analyzer (Siemens, Germany). Other biochemical tests, such as serum high-sensitivity CRP (hsCRP), were measured using an immunoturbidimetric assay, uric acid was measured using the uricase enzymatic colorimetric method, and serum creatinine was measured using the kinetic method on a Beckman Coulter AU480 analyzer. Leptin and N-terminal pro-brain natriuretic peptide (NT-proBNP) levels were measured using an enzyme immunoassay on a Siemens Immulite 1000 analyzer. Cystatin C was measured on Alisei Q.S. (Radim Diagnostics, Italy), an automated enzyme-linked immunosorbent assay (ELISA) analyzer using the Alkor-Bio (Russia) test kits. Thorough quality control protocols were implemented. For internal quality control, the samples were run for each batch. The instruments were regularly calibrated and maintained.

Cardiorenometabolic risk factors

The following primary and residual cardiorenometabolic risk factors were considered [4]: (1) abdominal obesity, assessed by WC ≥80 cm and/or WHR >0.85 for women, and ≥94 cm and/or WHR >0.9 for men; (2) elevated BP characterized by systolic BP (SBP) ≥130 mmHg and/or diastolic BP (DBP) ≥85 mmHg, or use of antihypertensive medication; (3) reduced HDL-c, defined as <1.3 mmol/l in women and <1.0 mmol/l in men, or use of lipid-lowering therapy; (4) fasting hypertriglyceridemia, indicated by a serum triglyceride level ≥1.7 mmol/l or use of lipid-lowering drugs; (5) hyperinsulinemia >27 µU/ml; (6) insulin resistance defined as confirmed when the Homeostasis Model Assessment of Insulin Resistance (HOMA-IR) value exceeded 2.52 [11]; (7) hyperleptinemia >11.1 ng/ml, suggestive of leptin resistance and adipocyte dysfunction; (8) elevated levels of NT-proBNP >125 pg/ml, indicating potential cardiac stress; (9) hyperuricemia >360 μmol/l; (10) non-HDL-c, calculated as total cholesterol minus HDL-c, with elevated levels considered >3.4 mmol/l [12]; (11) high-sensitivity C-reactive protein >2 mg/l was considered as having a higher risk of heart disease [13]; (12) visceral adiposity index estimated considering the age [14] and (13) elevated cystatin C >1.2 mg/l, indicating potential early renal impairment. Estimated glomerular filtration rate (eGFR) was calculated using various formulae and classified according to the Kidney Disease: Improving Global Outcomes (KDIGO) guidelines [8].

Echocardiographic assessment

To reduce bias, a team of two trained and certified cardiologists performed Doppler echocardiography on a Mindray DC-8 machine (Mindray Medical International Limited, Shenzhen, China), following strict protocols outlined in the guidelines [15]. These cardiologists underwent a standardized training program to ensure consistency in technique and interpretation. To further minimize variability, they were blinded to the clinical outcomes of the patients, which was crucial in maintaining the integrity of the results. We assessed the left atrial (LA) volume and size, left ventricular (LV) end-diastolic volume (EDV), end-systolic volume (ESV), ejection fraction (EF), and stroke volume (SV). Furthermore, we calculated the interventricular septal thickness, left ventricular posterior wall thickness, and myocardial mass by indexing to the body surface area.

Ethical approval

The study was approved by the local ethics committee of Kazan State Medical University (protocol #6, dated June 22, 2021).

Sample size calculation

The sample size, including for tertile-based comparisons, was determined while accounting for variables that do not follow a normal distribution. Assuming a medium effect size (Cohen’s d = 0.5), power = 80%, and two-sided α = 0.05, a total of 117 participants were required (39 participants per tertile). To account for the reduced efficiency in the nonparametric tests, this was inflated by 15%, yielding 135 participants (45 per tertile). Our enrollment of 169 participants ensured adequate power.

Statistical analysis

All statistical analyses were performed using IBM SPSS Statistics, version 27 (IBM Corp., Armonk, USA). The Kolmogorov-Smirnov test was used to assess normality. As the data were not normally distributed, nonparametric tests were employed. Descriptive statistics were used to obtain frequencies and percentages for categorical variables. Significant differences in categorical variables were tested using Pearson's chi-square test or Fisher's exact test. Continuous variables were presented as medians and interquartile ranges (IQRs: 25th-75th percentiles). The participants were stratified into sex-specific cystatin C tertiles based on the internal data distribution of the study cohort (sex-specific): tertile I (cystatin C ≤0.69 mg/l), tertile II (cystatin C = 0.70-0.84 mg/l), and tertile III (cystatin C ≥0.85 mg/l). The Mann-Whitney U test was used to compare two independent groups, while the Kruskal-Wallis test was employed for comparing three or more groups. Spearman's correlation analysis was performed to assess the relationship between variables. Separate linear regression analyses were conducted to identify key predictors of eGFR in female and male participants. The stepwise procedure selected variables from a pool that included all measured cardiorenometabolic parameters. Covariates such as age, smoking, or specific medications were not included in the models due to the study's focus on biomarker associations and to avoid overfitting with the available sample size. A two-tailed p-value of ≤0.05 was considered statistically significant.

## Results

Baseline characteristics of cardiorenometabolic risk factors

The study cohort comprised 169 participants (89 female participants and 80 male participants) with a median age of 34 years (IQR: 30-39 years). The prevalence of cardiorenometabolic risk factors is presented in Table 2. High body mass (BMI ≥25 kg/m²) was prevalent in the cohort, with no significant sex differences. However, abdominal obesity was more common in female participants. Body composition analysis did not reveal any sarcopenia. Sex-specific disparities were notable in relation to blood pressure: male participants exhibited higher rates of elevated systolic and diastolic blood pressure. Lipid abnormalities, such as hypertriglyceridemia, were frequent in male participants, while no significant sex differences were observed in other lipid profile parameters (p = 0.099-0.561). Lowering the HbA1c threshold from 6.0%-6.4% to 5.7%-6.4% nearly doubled the proportion of at-risk individuals (19.5% vs. 33.7%, respectively), reclassifying an additional 24 patients. In both cases, although statistically insignificant, a higher prevalence was observed among female participants. Renal parameters also demonstrated sex differences. Hyperuricemia and elevated cystatin C levels and albumin-to-creatinine ratio were significantly more common in male participants as compared to female participants (p = 0.033-0.003). Interestingly, no statistically significant sex differences existed in the eGFR (p = 0.227-0.848) at various thresholds. Hyperleptinemia and elevated levels of hsCRP, NT-proBNP, and heart rate were also markedly more common in female participants (p = 0.000-0.0021).

**Table 2:** Baseline characteristics of cardiorenometabolic risk factors n is the number of participants with deranged parameters, % is the proportion of subjects with deranged parameters presented as percentages and p-value denotes the significance obtained by Pearson’s chi-square or Fisher’s exact test. BMI: body mass index, WC: waist circumference, WHR: waist-to-hip ratio, SBP: systolic blood pressure, DBP: diastolic blood pressure, ↓HDL-c: low high-density lipoprotein cholesterol, LDL-c: low-density lipoprotein cholesterol, IFG: impaired fasting glucose, HOMA-IR: Homeostasis Model Assessment of Insulin Resistance, eGFR: estimated glomerular filtration rate, CRP: C-reactive protein, NT-proBNP: N-terminal pro-brain natriuretic peptide, CKD-EPI: Chronic Kidney Disease Epidemiology Collaboration

Parameter	Total (n = 169)		Female (n = 89)		Male (n = 80)		p-value	
	n (%)		n (%)		n (%)			
Anthropometry and bioimpedance analysis								
BMI ≥25 kg/m^2^	112 (66.3)	59 (66.3)		53 (66.3)				0.995
BMI ≥30 kg/m^2^	53 (31.4)	31 (34.9)		22 (27.5)				0.305
WC ≥80 cm in females; WC ≥94 cm in males	89 (52.7)	56 (62.9)		33 (41.3)				0.005
WHR >0.85 in females; WHR >0.90 in males	48 (28.4)	25 (28.0)		23 (28.8)				0.924
Abdominal obesity	93 (55.0)	56 (62.9)		37 (46.3)				0.030
Increased body fat percentage	28 (16.6)	11 (12.4)		17 (21.3)				0.129
Visceral fat level >12	8 (4.7)	3 (3.4)		5 (6.3)				0.379
Low muscle mass (sarcopenia)	0	0		0				0
Blood pressure								
SBP ≥130 mmHg	44 (26.0)		12 (13.5)		32 (40.0)		0.000	
DBP ≥85 mmHg	36 (21.3)		11 (12.4)		25 (31.3)		0.003	
Diagnosis of hypertension + patients on antihypertensive medication	5 (3.0)		1 (1.1)		4 (5.0)		0.215	
Lipid profile								
Total cholesterol ≥5 mmol/l	73 (43.2)		34 (38.2)		39 (48.8)	0.167		
Triglycerides ≥1.7 mmol/l	29 (17.2)		10 (11.2)		19 (23.8)	0.031		
↓HDL-c (in females, <1.2 mmol/l; in males, <1.0 mmol/l)	43 (25.4)		21 (23.6)		22 (27.5)	0.561		
LDL-c >3 mmol/l	98 (58.0)		49 (55.0)		49 (55.0)	0.415		
Non-HDL-c >3.4 mmol/l	88 (52.1)		41(46.0)		47 (58.8)	0.099		
Glycemic profile								
IFG	3 (1.8)		1(1.12)		2(2.5)	0.499		
HbA1c (5.7–6.4), %	57 (33.7)		35 (39.3)		22(27.5)	0.104		
HbA1c (6.0–6.4), %	33 (19.5)		18 (20.2)		15 (18.8)	0.809		
HOMA-IR >2.52	36 (21.3)		19 (21.4)		17 (21.3)	0.988		
Hyperinsulinemia >27 µU/ml	6 (3.6)		3(3.4)		3 (3.8)	0.894		
Renal profile								
Uric acid >360 µmol/l	19 (11.2)		4 (4.5)		15(18.8)	0.003		
Serum creatinine >124 µmol/l	1 (0.6)		0		1 (1.3)	0.290		
Cystatin C >1.2 mg/l	10 (5.9)		2 (2.2)		8 (10.0)	0.033		
Urine albumin:creatinine ratio ≥30 mg/g	7 (4.1)		0		7 (8.8)	0.005		
eGFR (CKD-EPI creatinine) ≥120 ml/min/1.73 m^2^	5 (3.0)		2 (2.2)		3 (3.8)	0.565		
eGFR (CKD-EPI creatinine) ≤90 ml/min/1.73 m^2^	37 (21.9)		20 (22.5)		16 (21.3)	0.848		
eGFR (CKD-EPI cystatin C) ≥120 ml/min/1.73 m^2^	45 (26.6)		22 (24.7)		23 (28.7)	0.554		
eGFR (CKD-EPI cystatin C) ≤90 ml/min/1.73 m^2^	13 (7.7)		6 (6.7)		7 (8.8)	0.625		
eGFR (CKD-EPI creatinine-cystatin) ≥120 ml/min/1.73 m^2^	20 (11.8)		8 (9.0)		12 (15.0)	0.227		
eGFR (CKD-EPI creatinine-cystatin) ≤90 ml/min/1.73 m^2^	9 (5.3)		4 (4.5)		5 (6.3)	0.612		
Other risk factors								
High-sensitivity CRP >2 mg/l	59 (34.9)		39 (43.8)		20 (25)	0.010		
Leptin >11.1 ng/ml	78 (46.2)		64 (71.9)		14 (17.5)	0.000		
NT-proBNP >125 ng/ml	26 (15.4)		21 (23.6)		5 (6.3)	0.002		
Heart rate >80 per minute	45 (26.6)		32 (36.0)		13 (16.3)	0.004		

The median values and interquartile ranges for the anthropometric, metabolic, and biochemical parameters are presented in Table 3. Female participants had a significantly higher body fat percentage, while male participants demonstrated a larger waist circumference and higher waist-to-hip ratio. Sex-specific differences in blood pressure were evident, with male participants showing higher median systolic and diastolic pressures. Lipid profiles revealed that male participants had higher triglycerides and lower HDL cholesterol. Renal markers such as uric acid and cystatin C were elevated in male participants. Female participants exhibited higher median leptin and NT-proBNP levels.

**Table 3:** Median values of cardiorenometabolic parameters n is the number of participants in a particular group, Me is the median (interquartile range, 25th–75th percentile), and p-value is obtained from the Mann-Whitney U test. BMI: body mass index, WC: waist circumference, WHR: waist-to-hip ratio, SBP: systolic blood pressure, DBP: diastolic blood pressure, HDL-c: high-density lipoprotein cholesterol, LDL-c: low-density lipoprotein cholesterol, HOMA-IR: Homeostasis Model Assessment of Insulin Resistance, eGFR: estimated glomerular filtration rate, CRP: C-reactive protein, NT-proBNP: N-terminal pro-brain natriuretic peptide, CKD-EPI: Chronic Kidney Disease Epidemiology Collaboration

Parameter	Total (n = 169)	Female (n = 89)	Male (n = 80)	p-value
	Me (25%–75%)	Me (25%–75%)	Me (25%–75%)	
Age	34.00 (30.00-39.00)	35.00 (30.00-39.00)	34.00 (29.30-38.00)	0.423
Anthropometry and bioimpedance analysis				
BMI, kg/m^2^	26.7 (23.85-30.5)	26.60 (23.60-32.55)	26.75 (24.02-30.17)	0.548
WC, cm	87.00 (79.25-97.25)	83.00 (76.00-96.00)	91.00 (83.00-98.00)	0.003
WHR	0.840 (0.78-0.90)	0.80 (0.73-0.86)	0.87 (0.83-0.91)	0.000
Body fat percentage, %	28.50 (21.10-37.55)	36.80 (30.85-41.75)	21.70 (16.67-25.07)	0.000
Visceral fat level	7.00 (4.00-9.00)	6.00 (4.00-8.00)	7.00 (5.00-9.00)	0.083
Visceral adiposity index	1.07 (0.73-1.63)	1.03 (0.73-1.58)	1.23 (0.73-1.80)	0.367
Blood pressure				
SBP, mmHg	121.00 (112.00-130.00)	115.00 (106.50-122.00)	127.00 (121.00-134.00)	0.000
DBP, mmHg	76.00 (70.00-82.00)	72.00 (67.00-80.00)	80.00 (72.25-87.00)	0.000
Lipid profile				
Total cholesterol, mmol/l	4.89 (4.16-5.49)	4.72 (4.16-5.18)	4.98 (4.14-5.85)	0.221
Triglycerides, mmol/l	0.92 (0.64-1.38)	0.84 (0.61-1.13)	1.06 (0.73-1.67)	0.004
HDL-c, mmol/l	1.33 (1.10-1.53)	1.42 (1.20-1.61)	1.18 (1.00-1.36)	0.000
LDL-c, mmol/l	3.18 (2.54-3.70)	3.13 (2.52-3.61)	3.31 (2.56-3.90)	0.131
Non-HDL-c, mmol/l	3.48 (2.82-4.2)	3.32 (2.76-3.89)	3.75 (2.89-4.64)	0.025
Glycemic profile				
Glucose, mmol/l	4.3 (3.91-4.60)	4.3 (3.90-4.60)	4.25 (3.91-4.6)	0.820
Glycated hemoglobin (HbA1c), %	5.20 (4.92-5.70)	5.50 (5.10-5.9)	5.20 (4.92-5.70)	0.012
Insulin, μIU/ml	8.43 (4.70-13.40)	7.46 (4.89-12.50)	8.43 (4.70-13.40)	0.596
HOMA-IR	1.49 (0.87-2.43)	1.41 (0.87-2.33)	1.66 (0.84-2.48)	0.572
Renal profile				
Uric acid, μmol/l	311.75 (263.63-369.50)	266.76 (235.64-309.46)	364.23 (323.80-401.95)	0.000
Cystatin C, mg/l	0.77 (0.67-0.86)	0.71 (0.65-0.82)	0.81 (0.70-0.90)	0.000
Creatinine, μmol/l	80.46 (69.32-89.89)	69.75 (65.58-77.17)	88.58 (81.77-96.98)	0.000
Urine Albumin:creatinine ratio, mg/g	10.00 (3.00-15.00)	10.00 (3.00-15.00)	10.00 (3.0-15.00)	0.874
eGFR (CKD-EPI creatinine), ml/min/1.73 m^2^	100.00 (90.00-110.00)	98.00 (90.00-109.50)	101.00 (90.00-111.0)	0.431
eGFR (CKD-EPI cystatin C), ml/min/1.73 m^2^	114.00 (101.00-121.00)	115.00 (103.00-120.00)	112.00 (99.00-122.00)	0.621
eGFR (CKD-EPI creatinine-cystatin), ml/min/1.73 m^2^	109.00 (99.00-115.00)	109.00 (99.00-115.50)	108.00 (99.00-114.00)	0.785
Other risk factors				
High-sensitivity CRP, mg/l	1.03 (0.54-2.11)	1.69 (0.70-4.13)	1.03 (0.54-2.11)	0.009
Leptin, ng/ml	12.47 (5.39-28.19)	26.60 (16.50-43.19)	5.39 (2.91-9.34)	0.000
NT-proBNP, ng/ml	66.70 (44.30-105.50)	92.30 (60.10-129.25)	51.40 (34.50-78.97)	0.000
Heart rate, per minute	73.00 (68.00-80.00)	74.00 (69.50-83.50)	72.00 (67.25-77.00)	0.014

These findings underscore the significant sex-based variations in cardiorenometabolic risk profiles, particularly in adiposity distribution, blood pressure, and lipid metabolism. This pattern of sex-based variation was also evident for NT-proBNP, with female participants exhibiting significantly higher median levels than male participants (92.30 ng/ml vs. 51.40 ng/ml, p < 0.001). 

Given the significant sex difference in the prevalence of elevated cystatin C (10.0% in male participants vs. 2.2% in female participants, p = 0.033) and the distinct cardiometabolic risk profiles, we stratified the analysis by both sex and cystatin C tertile. This approach allowed us to investigate the interaction between sex and varying levels of cystatin C (as a continuous gradient) on cardiorenometabolic parameters, to determine if the relationship between cystatin C and other risk factors differed by sex.

Sex-stratified cardiorenometabolic profiles across cystatin C tertiles

Stratification by sex and cystatin C tertiles revealed a distinct pattern in cardiorenometabolic risk factors (Table 4). Among female participants, higher cystatin C tertiles were associated with increasing BMI (p-trend = 0.010), waist circumference (p-trend = 0.039), visceral fat levels (p-trend = 0.008), glucose (p-trend = 0.012), hsCRP (p-trend = 0.011), and leptin (p-trend = 0.048). However, not all parameters showed linear trends; for instance, WHR demonstrated a significant sex-by-tertile interaction (p = 0.003) primarily driven by the consistently and significantly higher absolute values in male participants across all tertiles, rather than a strong graded increase within female participants. Notably, a statistically significant decline in eGFR (CKD-EPI cystatin C) was observed in both female and male participants (p-trend < 0.001). However, apart from this finding, no significant trends were observed among male participants.

**Table 4:** Cardiorenometabolic risk factors stratified by sex and cystatin C tertiles n is the number of participants in a particular group. Data are presented as median (Me) (interquartile range, 25th–75th percentile) for continuous variables across cystatin C tertiles. p-trend is calculated using the Kruskal-Wallis test for non-normally distributed continuous variables. p-interaction is derived from general linear model (GLM) testing the interaction between sex and cystatin C tertiles. BMI: body mass index, WC: waist circumference, WHR: waist-to-hip ratio, SBP: systolic blood pressure, DBP: diastolic blood pressure, HDL-c: high-density lipoprotein cholesterol, LDL-c: low-density lipoprotein cholesterol, HOMA-IR: Homeostasis Model Assessment of Insulin Resistance, eGFR: estimated glomerular filtration rate, CRP: C-reactive protein, NT-proBNP: N-terminal pro-brain natriuretic peptide, CKD-EPI: Chronic Kidney Disease Epidemiology Collaboration

Variable	Cystatin C tertiles			p-trend (overall)	p-interaction (sex x tertile)
	Tertile I (cystatin C ≤0.69 mg/l), n = 57	Tertile II (cystatin C = 0.70–0.84 mg/l), n = 61	Tertile III (cystatin C ≥0.85 mg/l), n = 51		
Age					
Female	35.00 (31.25-40.00)	35.00 (30.25-37.00)	35.50 (31.00-39.50)	0.860	0.731
Male	32.50 (28.50-35.50)	35.50 (31.00-39.00)	35.00 (29.50-37.25)	0.296	
Anthropometry and bioimpedance analysis					
BMI, kg/m^2^					
Female	25.00 (22.72-28.12)	30.45 (23.97-34.37)	27.25 (24.42-38.27)	0.010	0.015
Male	26.20 (24.70-28.72)	26.20 (23.75-29.90)	26.45 (23.22-30.35)	0.995	
WC, cm					
Female	81.00 (72.50-86.75)	89.50 (77.75-102.50)	81.50 (76.12-104.25)	0.039	0.027
Male	89.25 (84.50-94.00)	90.00 (84.00-96.00)	91.00 (80.87-98.50)	0.967	
WHR					
Female	0.78 (0.72-0.83)	0.82 (0.73-0.90)	0.76 (0.70-0.82)	0.159	0.003
Male	0.87 (0.84-0.92)	0.86 (0.82-0.89)	0.88 (0.84-0.93)	0.302	
Body fat percentage, %					
Female	35.00 (29.95-39.52)	39.35 (32.65-45.32)	37.25 (32.70-45.67)	0.071	0.000
Male	20.30 (16.10-24.10)	21.70 (17.07-25.00)	20.90 (14.37-22.97)	0.569	
Visceral fat level					
Female	5.00 (3.25-7.00)	7.00 (4.25-9.75)	7.00 (4.25-11.75)	0.008	0.010
Male	6.50 (4.50-9.00)	7.50 (5.00-9.00)	7.00 (3.00-10.00)	0.520	
Visceral adiposity index					
Female	0.98 (0.67-1.59)	1.13 (0.78-1.57)	0.88 (0.68-1.42)	0.345	0.787
Male	1.30 (0.73-1.43)	1.04 (0.68-1.76)	1.33 (0.72-2.68)	0.978	
Blood pressure					
SBP, mmHg					
Female	114.50 (107.00-122.50)	116.00 (105.00-124.00)	112.00 (102.75-116.50)	0.372	0.000
Male	127.50 (120.50-131.75)	127.00 (121.00-133.75)	129.00 (118.75-135.00)	0.771	
DBP, mmHg					
Female	72.00 (67.25-79.00)	75.00 (68.00-86.25)	71.00 (63.25-79.75)	0.178	0.001
Male	80.50 (72.50-85.50)	80.00 (75.00-87.00)	81.00 (72.00-90.00)	0.910	
Lipid profile					
Total cholesterol, mmol/l					
Female	4.77 (4.14-5.17)	4.77 (3.72-5.15)	4.57 (4.19-5.05)	0.933	0.356
Male	4.34 (3.98-5.01)	5.15 (4.74-5.89)	5.10 (3.77-5.81)	0.174	
Triglycerides, mmol/l					
Female	0.82 (0.56-1.04)	0.84 (0.62-1.27)	0.75 (0.53-1.13)	0.652	0.179
Male	0.97 (0.80-1.26)	1.09 (0.71-1.77)	1.09 (0.76-2.15)	0.642	
HDL-c, mmol/l					
Female	1.42 (1.20-1.66)	1.40 (1.10-1.63)	1.43 (1.24-1.57)	0.703	0.001
Male	1.13 (0.96-1.34)	1.26 (1.10-1.41)	1.16 (0.99-1.33)	0.159	
LDL-c, mmol/l					
Female	2.15 (2.53-3.63)	3.14 (2.24-3.72)	2.90 (2.44-3.39)	0.824	0.068
Male	2.90 (2.52-3.34)	3.63 (2.90-4.27)	3.27 (2.21-3.85)	0.088	
Non-HDL-c, mmol/l					
Female	3.35 (2.76-3.91)	3.40 (2.57-4.16)	3.17 (2.73-3.53)	0.865	0.159
Male	3.19 (2.85-3.75)	3.81 (3.26-4.73)	3.75 (2.44-4.68)	0.301	
Glycemic profile					
Glucose, mmol/l					
Female	4.04 (3.72-4.47)	4.45 (4.02-4.80)	4.40 (4.11-4.85)	0.012	0.300
Male	4.20 (3.90-4.82)	4.25 (4.00-4.60)	4.30 (3.90-4.60)	0.995	
Glycated hemoglobin (HbA1c), %					
Female	5.50 (5.12-5.90)	5.20 (5.10-5.70)	5.55 (5.12-6.00)	0.559	0.229
Male	5.20 (5.02-5.60)	5.15 (4.90-5.85)	5.25 (4.90-5.82)	0.881	
Insulin, μIU/ml					
Female	6.39 (4.68-10.72)	9.52 (5.21-13.27)	7.83 (5.15-11.00)	0.212	0.230
Male	7.82 (4.76-10.55)	8.88 (5.52-14.62)	6.77 (4.16-11.65)	0.200	
HOMA-IR					
Female	1.16 (0.82-2.05)	1.89 (0.88-3.11)	1.49 (1.10-2.02)	0.236	0.194
Male	1.60 (0.71-2.51)	1.90 (0.99-3.37)	1.26 (0.72-2.27)	0.215	
Renal profile					
Uric acid, μmol/l					
Female	244.83 (223.93-279.79)	273.46 (257.71-329.94)	265.17 (235.21-338.12)	0.051	0.000
Male	331.48 (306.74-372.94)	338.02 (331.93-385.96)	375.56 (328.80-438.10)	0.321	
Urine albumin:creatinine ratio, mg/g					
Female	10.00 (5.00-15.00)	5.00 (5.00-15.00)	5.00 (3.00-10.00)	0.299	0.232
Male	10.00 (3.00-10.00)	10.00 (3.00-15.00)	10.00 (3.00-15.00)	0.540	
eGFR (CKD-EPI cystatin C), ml/min/1.73 m^2^					
Female	120.50 (116.75-126.00)	111.00 (104.50-113.00)	91.00 (86.00-96.50)	0.000	0.000
Male	128.50 (127.00-135.00)	117.00 (114.00-122.00)	97.00 (90.75-102.50)	0.000	
Other risk factors					
High-sensitivity CRP, mg/l					
Female	1.05 (0.61-2.52)	2.13 (1.05-5.55)	4.28 (0.72-8.52)	0.011	0.000
Male	0.88 (0.34-3.36)	1.19 (0.58-2.11)	0.99 (0.53-1.96)	0.842	
Leptin, ng/ml					
Female	21.40 (14.88-29.52)	34.99 (22.68-48.33)	31.39 (16.67-53.00)	0.048	0.000
Male	5.39 (3.46-13.13)	6.44 (4.13-11.67)	4.71 (2.07-9.16)	0.791	
NT-proBNP, ng/ml					
Female	95.55 (59.80-127.25)	81.65 (57.77-131.00)	99.20 (66.20-141.25)	0.826	0.048
Male	55.15 (34.35-95.52)	50.85 (34.05-66.57)	53.55 (38.00-80.82)	0.846	
Heart rate, per minute					
Female	77.50 (69.75-84.25)	74.50 (71.50-83.50)	72.00 (66.50-80.50)	0.271	0.011
Male	72.00 (68.00-79.00)	71.00 (67.00-75.00)	73.00 (66.75-78.00)	0.557	

Key sex-specific interactions (p-interaction <0.05) were identified for BMI, WC, WHR, body fat percentage, visceral fat level, blood pressure, HDL cholesterol, uric acid, eGFR (CKD-EPI cystatin C), hsCRP, leptin, NT-proBNP, and heart rate. These findings underscore sex-specific vulnerabilities in cardiorenometabolic risk profiles, even in early adulthood, and emphasize the need for tailored risk assessment strategies. Due to the exploratory nature of the analyses involving multiple comparisons, no statistical correction (e.g., Bonferroni) was applied. Therefore, the results, particularly the numerous interaction effects, should be interpreted with caution, serving as a basis for generating hypotheses for future validation, while acknowledging an increased risk of false-positive findings.

Echocardiographic parameters and cystatin C tertiles

In the sex-stratified analysis of echocardiographic parameters across cystatin C tertiles, no significant linear trends (p-trend range = 0.967-0.160) were observed for most variables in either sex. This lack of a clear trend may be due to the relatively young age and preserved cardiac function of the cohort, where subclinical changes in structure may not yet linearly correlate with early renal markers, or due to limited statistical power to detect subtle associations within tertiles. However, significant interactions between sex and cystatin C tertiles (p-interaction ≤0.05) were evident for parameters such as interventricular septal thickness, posterior wall thickness, left ventricular mass, end-diastolic dimension, and end-diastolic volume. These interactions suggest that the relationship between cystatin C levels and cardiac structural/functional parameters differs between sexes, likely reflecting inherent baseline differences in echocardiographic reference values (e.g., higher left ventricular mass and larger chamber dimensions in male participants). Further studies are needed to explore these sex-dependent associations in larger cohorts. Due to the lack of significant trends across tertiles and the complexity of interactions, we omitted this detailed stratification from the main manuscript.

Correlation analysis

Spearman’s rank correlation analysis revealed sex-specific associations between cystatin C and cardiometabolic and echocardiographic parameters. In female participants, cystatin C demonstrated significant positive correlations with multiple indices, including body mass index, waist circumference, body fat percentage, visceral fat level, leptin, and CRP. Notably, no significant correlations were observed between cystatin C levels and cardiometabolic parameters in male participants. In contrast, serum creatinine levels exhibited broader associations with these parameters in male participants. However, the eGFR derived from cystatin C in female participants and creatinine in male participants demonstrated stronger and more extensive correlations with cardiometabolic and echocardiographic parameters than their respective individual biomarkers.

**Figure 1:**
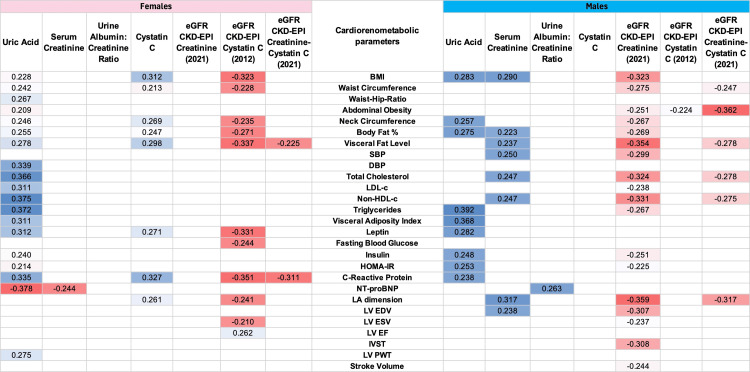
Correlation matrix showing the most significant correlations among cardiorenometabolic and echocardiographic variables This heat map shows the Spearman's rho for parameters with significant (p ≤ 0.05) correlations. eGFR: estimated glomerular filtration rate, BMI: body mass index, SBP: systolic blood pressure, DBP: diastolic blood pressure, LDL-c: low-density lipoprotein cholesterol, non-HDL-c: non-high-density lipoprotein cholesterol, HOMA-IR: Homeostasis Model Assessment of Insulin Resistance, NT-proBNP: N-terminal pro-brain natriuretic peptide, LA: left atrium, LV EDV: left ventricular end-diastolic volume, LV ESV: left ventricular end-systolic volume, LV EF: left ventricular ejection fraction, IVST: interventricular septal thickness, LV PWT: left ventricular posterior wall thickness

For instance, in female participants, while cystatin C correlated only with left atrial dimension, eGFR_cysC _showed inverse associations with LA dimension, left ventricular end-systolic volume, and positive associations with left ventricular ejection fraction. Similarly, in male participants, eGFR_creatinine _correlated inversely with LA dimension, LV EDV, LV ESV, LV stroke volume, and interventricular septal thickness. A comprehensive visualization of these relationships is provided in the correlation matrix heatmap (Figure 1).

Linear regression analysis

Given the sex-specific patterns observed in the correlation analysis, we conducted separate linear regression analyses for female and male participants using eGFR_cystatinC_ and eGFR_creatinine_, respectively. This approach aimed to evaluate independent associations between cardiorenometabolic variables while accounting for cardiorenal syndrome and metabolic, hemodynamic, and neurohumoral factors (Table 5). The selection of these specific eGFR formulae was data-driven, as the correlation analysis revealed stronger and more extensive correlations with cardiometabolic parameters in their respective sexes. This method allowed us to identify the most sensitive marker of cardiorenometabolic crosstalk for each sex, rather than directly comparing the two biomarkers within the same model.

**Table 5:** Linear regression results for eGFR by sex Separate stepwise linear regression models were constructed for female participants (eGFR based on cystatin C; model p < 0.001) and male participants (eGFR based on creatinine; model p = 0.029). Dashes (—) denote variables not included in the respective sex-specific model. CRP: C-reactive protein, SBP: systolic blood pressure

Variables	Females (eGFR_cystatinC_)			Males (eGFR_creatinine_)		
	B	SE	p-value	B	SE	p-value
(Constant)	73.863	13.056	<0.001	110.289	3.081	<0.001
High-sensitivity CRP, mg/l	-1.470	0.416	<0.001	—	—	—
Visceral fat level	-1.506	0.435	<0.001	-2.329	0.615	<0.001
SBP, mmHg	0.421	0.120	<0.001	—	—	—
Insulin, μU/ml	0.259	0.113	0.024	—	—	—
Leptin, ng/ml	—	—	—	0.807	0.362	0.029
Adjusted R^2^	0.283			0.166		

In female participants, a stepwise linear regression analysis using eGFR based on cystatin C identified significant associations between cardiometabolic markers and renal function. Higher hsCRP levels and visceral fat were inversely associated with eGFR, while systolic blood pressure and insulin showed positive relationships. The model (p < 0.001) explained 28.3% of the variance in eGFR, highlighting the combined influence of inflammation, adiposity, and metabolic factors on renal function in female participants.

In male participants, the regression model using eGFR based on creatinine revealed visceral fat level as a strong inverse predictor, while leptin exhibited a positive association. The model (p = 0.029) explained 16.6% of the variance, thereby emphasizing sex-specific differences. Notably, the role of leptin in male participants contrasted with the prominence of hsCRP and insulin in female participants, suggesting distinct biological pathways linking metabolic health to kidney function between sexes.

## Discussion

Our study focused on assessing sex differences in the early stages of the cardiorenometabolic continuum among young adults with risk factors but no overt disease. It revealed marked sex-specific variations in the prevalence and patterns of cardiorenometabolic risk factors, along with differing associations between cystatin C levels and cardiac, renal, and metabolic parameters.

Women exhibited higher rates of abdominal obesity than men, which is in line with previous results that highlighted sex-specific patterns in adiposity distribution. This higher prevalence, despite a similar overall BMI may be influenced by hormonal factors [16]. Notably, despite a higher prevalence of abdominal obesity, and the higher median values of body fat percentage in female participants, the frequency of increased visceral fat level and body fat percentage in female and male participants did not differ statistically. However, despite the similar prevalence of elevated visceral fat (level >12) between groups, the continuous measure of visceral fat level emerged as a strong independent predictor of renal function in regression models for both sexes. This indicates that even within the normal-to-moderate range, variations in visceral adiposity are critically linked to early renal changes, underscoring its value as a continuous risk factor beyond a simple dichotomous threshold. These findings underscore the importance of measuring body fat percentage and visceral fat levels, rather than relying solely on abdominal obesity criteria to get a clearer picture. Interestingly, our study also found a higher leptin disparity between sexes, with female participants showing median leptin levels nearly five times higher than those in male participants. This substantial difference, which was not adjusted for the significantly higher body fat percentage in female participants, likely reflects the combined effect of greater adiposity and potential adipocyte dysfunction [17]. The exaggerated leptin disparity in our cohort may reflect enhanced adipocyte leptin production in young adult women, potentially driven by estrogen-mediated mechanisms [18]. It is important to underscore that pronounced hyperleptinemia raises concerns about early-onset leptin resistance, which could contribute to the observed metabolic dysregulation in female participants [18].

Young male participants in our study had a higher prevalence and higher median values of increased SBP and/or DBP, which is consistent with other findings and may be attributed to a combination of sex-specific physiological differences, as well as metabolic, behavioral, and other risk factors [19]. Similarly, they exhibited a higher prevalence of hypertriglyceridemia as well as higher median levels. The sex-specific dyslipidemia patterns observed in our young adult cohort mirror those typically observed in older populations, suggesting that these metabolic divergences manifest early in adulthood [20].

Furthermore, other findings include a higher prevalence of elevated high-sensitivity CRP levels, along with increased levels of NT-proBNP and elevated heart rate in females. These findings align with results obtained by Lakunchykova et al. in Russian women, suggesting a significant association between obesity-related inflammation, cardiovascular stress, and higher heart damage due to non-ischemic pathways in women [21]. The presence of these markers may reflect underlying metabolic dysregulation, which contributes to the heightened cardiovascular risk [16]. Consequently, these findings necessitate targeted interventions to improve health outcomes in women. While the absolute numerical differences for some parameters (e.g., HDL-c, NT-proBNP, heart rate) were modest and may lack immediate clinical actionability in this cohort of young adults, their statistical significance highlights early diverging pathophysiological trends between sexes that warrant attention in longitudinal studies.

Renal parameters also exhibited notable sex differences. Male participants showed higher rates of hyperuricemia, elevated cystatin C levels, and increased albumin-to-creatinine ratios. These findings align with previous studies reporting higher serum uric acid levels and albuminuria in men. The differences may be attributed to lower estrogen levels in men, which is believed to have a uricosuric effect. The increased albumin-to-creatinine ratios are likely multifactorial, involving behavioral and metabolic risk factors, as well as early kidney dysfunction [22,23]. Interestingly, elevated cystatin C levels in men, despite comparable estimated GFR across sexes, suggest potential sex-specific variations in cystatin C synthesis or excretion that warrant further investigation.

Based on the significant sex differences in the prevalence of cystatin C (10.0% in male participants vs. 2.2% in female participants, p = 0.033) and its emerging role in cardiometabolic risk beyond renal function, we decided to study the cardiorenometabolic and echocardiographic parameters considering cystatin C tertile-based stratification. Apart from being a more reliable parameter for estimating GFR, cystatin C correlates with subclinical inflammation and endothelial dysfunction, by various cardiometabolic pathways that may differ by sex [24,25]. Its production is constant and less influenced by muscle mass than creatinine, making it potentially more reliable in populations with varying body composition. In the context of early cardiorenometabolic changes, cystatin C may serve as an integrative marker, linking adiposity-driven metabolic dysregulation (e.g., via inflammatory cytokines that can stimulate its production) with initial renal impairment, thereby reflecting broader system-level perturbations. Finally, by stratifying participants into tertiles, we aimed to elucidate graded associations between cystatin C levels and cardiorenometabolic risk, capturing early pathological trends that linear analyses might overlook.

Our stratification by sex and cystatin C tertiles revealed distinct patterns in cardiorenometabolic risk factors. The preceding discussion highlighted a higher prevalence of abdominal obesity, hsCRP, and leptin in women. Upon stratification, it was observed that these changes are associated with an increase in cystatin C, underscoring stronger associations with heightened inflammation and metabolic dysregulation in conjunction with renal dysfunction, thereby indicating a cardiorenometabolic association [16,26-27]. This is in line with our results of correlation analyses. The regression models further clarified these relationships. In female participants, eGFR based on cystatin C was independently predicted by hsCRP, visceral fat, systolic blood pressure, and insulin. This model highlights a network of inflammation, adiposity, and insulin resistance driving renal decline, a pathway increasingly recognized in cardiorenometabolic syndrome [2]. Male participants exhibited consistently higher absolute blood pressure values, while females showed a distinct pattern of SBP/DBP fluctuation across tertiles, with declining SBP/DBP in tertile III. This highlights sex-specific heterogeneity in the relationship between cystatin C and blood pressure regulation. The observed decline in SBP/DBP among female participants in the highest cystatin C tertile is intriguing and could potentially reflect early autonomic dysregulation or maladaptive vascular remodeling associated with advanced metabolic dysfunction. However, as autonomic tone and endothelial function were not directly measured, this interpretation remains speculative and requires validation in studies equipped with these measures. In male participants, the regression model using eGFR based on creatinine revealed visceral fat level as a strong inverse predictor, while leptin exhibited a positive association. The positive association between leptin and eGFR in males may be non-causal and instead reflect confounding by body composition. In this cross-sectional study, higher leptin levels likely indicate greater overall adiposity. Individuals with more adiposity may also have relatively preserved muscle mass, resulting in higher creatinine production and consequently a higher creatinine-based eGFR. This association might reverse in populations with overt kidney disease, where leptin clearance is reduced. The positive correlation with eGFR could indirectly indicate better preserved muscle mass (which correlates with creatinine production and higher eGFR) in individuals with higher overall adiposity. Alternatively, it may suggest a state of early leptin resistance where the hormone's metabolic effects are decoupled from its primary role in energy balance [28]. This finding highlights the need to interpret leptin levels within the broader context of body composition.

The difference in eGFR trajectories between sexes aligns with previous research and points to distinct underlying causes of kidney damage; for instance, women might be facing inflammation-related glomerular damage, whereas men seem to experience a quicker loss of kidney structure [2,8,26]. Furthermore, the interaction effects on blood pressure, HDL cholesterol, and heart rate underscore sex-specific cardiovascular responses to increasing cystatin C levels. For example, women in the highest tertile demonstrated unexpected decreases in both systolic and diastolic blood pressure, which might be attributed to autonomic dysregulation, endothelial dysfunction, or vascular remodeling. In contrast, men continued to show elevated blood pressures, aligning with their inherently higher baseline cardiovascular risk [29]. The general linear model (GLM) analysis for interaction effects did not adjust for potential confounding variables such as age, physical activity, or medication use.

Worth noting is the consistent behavior of NT-proBNP. The higher frequency, and median values both in the general cohort as well as in the sex-stratified cystatin C tertiles are in line with other findings and are likely to be driven by factors such as cardiovascular stress, inflammation, hyperinsulinemia and insulin resistance, hormonal modulation, as well as its production and clearance [30].

The significant sex interactions observed for echocardiographic parameters, even in the absence of linear trends, suggest that the pathophysiological link between early renal markers and cardiac morphology may be fundamentally different between men and women. Beyond inherent baseline differences in cardiac size, these interactions could reflect sex-specific patterns of end-organ response to metabolic stress. For instance, women may be predisposed to different types of subclinical remodeling (e.g., related to inflammation or diastolic function) in response to cardiorenometabolic risk, while men might exhibit changes more closely tied to hemodynamic load [29]. This warrants further investigation in larger longitudinal studies.

While guidelines recommend using eGFR based on both creatinine and cystatin C, our results underscore the importance of adopting sex-specific strategies for assessing and managing cardiorenometabolic risk in young adults with early renal dysfunction. For young women, markers such as cystatin C and eGFR_cysC_ may be particularly useful in detecting subclinical renal dysfunction, enabling timely preventive interventions. In contrast, for young men, eGFR based on creatinine and evaluation of visceral fat level and leptin may offer more relevant insights, even in the absence of overt metabolic disease.

The observed sex-specific associations may be attributed to distinct biological mechanisms. In women, estrogen influences body fat distribution, inflammatory tone, and renal hemodynamics, potentially creating an axis where cystatin C integrates adiposity-driven inflammation with early renal changes. In men, the stronger association between creatinine-based eGFR and visceral fat may be driven by androgen patterns that promote central adiposity and different pathways of metabolic renal crosstalk, potentially involving earlier vascular perturbations. These hypotheses require direct testing in future mechanistic studies.

From a public health perspective, our findings suggest that early risk stratification in young adults could be enhanced by adopting sex-specific approaches: prioritizing screening for inflammation and cystatin C in young women with adiposity, and focusing on blood pressure, visceral fat, and creatinine in young men. This could help identify at-risk individuals for targeted lifestyle interventions before overt disease develops.

Strengths and limitations

Key strengths of this study include the focus on a young adult population, a demographic often overlooked in cardiorenometabolic research, and the comprehensive assessment of traditional and emerging risk factors. Sex-stratified analyses and the use of regression analysis to capture heterogeneous associations further enhance the novelty of this work. Standardized protocols for biochemical and echocardiographic measurements minimized measurement bias. Although echocardiography was performed by certified cardiologists using standardized protocols, the study did not formally assess inter-observer variability, which is a potential source of measurement error, particularly for volumetric parameters. Finally, the exclusion of participants with overt cardiometabolic diseases ensured that observed associations reflect subclinical pathways, enhancing the relevance of findings to primary prevention.

This study has several limitations. First, its cross-sectional design precludes causal inferences; longitudinal cohorts are needed to establish temporal relationships between cystatin C and cardiorenometabolic outcomes. Second, the generalizability of our findings is limited by the single-center, convenience sampling strategy and the cohort's lack of ethnic diversity. Third, the unadjusted analyses mean that residual confounding by factors like lifestyle, genetics, or female hormonal status cannot be ruled out. Finally, the reliance on tertiles for cystatin C stratification, while statistically robust, may not reflect clinically validated cutoffs.

Future directions

First, future research should prioritize longitudinal studies to unravel the complex relationships between cystatin C, body composition, and cardiorenometabolic health in young adults. This approach will provide valuable insights into the temporal dynamics of these associations. Second, efforts should focus on validating the utility of cystatin C as a predictive biomarker for subclinical cardiac remodeling (e.g., left ventricular hypertrophy) to enhance risk stratification. This step is crucial before moving forward with interventional trials. Third, research should aim to establish or validate clinically relevant cystatin C thresholds specifically for young adult populations. This will improve the clinical utility of this biomarker for risk stratification in this age group. Fourth, studies should explore the intricate interplay of inflammatory processes and sex-specific hormones, such as leptin signaling and the balance between estrogen and androgens. This may elucidate the observed differences between men and women. Fifth, to ensure broader applicability, these findings should be validated across various ethnic groups and in individuals with existing cardiorenometabolic conditions. Finally, once the above-mentioned priorities have been addressed, interventional trials testing sex-tailored lifestyle or pharmacological strategies to mitigate early cardiorenometabolic dysfunction can be initiated. These trials will be essential for generating a solid evidence base and guiding physicians in managing these patients.

## Conclusions

This study underscores profound sex differences in cardiorenometabolic risk profiles among young adults, with female participants exhibiting adiposity-driven inflammation and male participants displaying early renal and vascular perturbations. Our findings suggest that cystatin C and creatinine may serve as sensitive markers of cardiometabolic-renal crosstalk, with associations indicating that cystatin C-based eGFR and creatinine-based eGFR might better capture risks in women and men, respectively. However, the utility of these markers for routine screening in young adults and the development of sex-optimized assessment protocols require further validation in larger, longitudinal studies to establish causality and clinical efficacy. These findings advocate for considering sex-stratified approaches in primary care, emphasizing early screening for abdominal obesity, inflammation, and renal dysfunction. Integrating novel biomarkers like cystatin C into risk assessment in research settings could help facilitate personalized lifestyle or pharmacological interventions, potentially attenuating the progression of cardiorenometabolic diseases in young adulthood. A focus on early detection and risk stratification is paramount for refining primary prevention strategies.
